# Assessing Barriers to Cancer Screening and Early Detection in Older Adults in Saudi Arabia: A Mixed-Methods Approach to Oncology Nursing Practice Implications

**DOI:** 10.3390/curroncol31120580

**Published:** 2024-12-08

**Authors:** Abdulaziz M. Alodhialah, Ashwaq A. Almutairi, Mohammed Almutairi

**Affiliations:** 1 Department of Medical Surgical Nursing, College of Nursing, King Saud University, Riyadh 11451, Saudi Arabia; mohalmutairi@ksu.edu.sa; 2 School of Nursing & Midwifery, Monash University, Melbourne, VIC 3004, Australia; ashwaq.almutairi@monash.edu

**Keywords:** cancer screening, older adults, barriers, mixed methods, accessibility, psychological factors

## Abstract

Background: Access to cancer screening services is crucial for early detection and improved survival rates, yet older adults in Saudi Arabia face significant barriers. Recent data from the Saudi Health Ministry indicate that cancer incidence in this demographic is rising, underscoring the urgent need for enhanced screening efforts. This study explores the factors influencing cancer screening behaviors among older adults in Riyadh, using a mixed-methods approach to identify and address these barriers effectively. Methods: The study integrated quantitative data from 100 participants aged 60 and above who attended King Saud University-affiliated healthcare centers, and qualitative insights from 20 semi-structured interviews. The Barriers to Cancer Screening Scale (BCSS) quantitatively assessed barriers, while the thematic analysis of interview data helped identify key themes. Results: Findings revealed significant barriers, categorized into three primary themes: accessibility challenges, psychological barriers, and social influences. These include logistical difficulties related to transportation and service availability, fears and anxieties regarding cancer diagnoses, and a lack of family support and cultural stigma, all of which impact participants’ willingness to engage in screening. Conclusion: The study underscores the multifaceted barriers faced by older adults in accessing cancer screening in Saudi Arabia. Tailored interventions that address logistical, psychological, and social factors are essential to enhance screening uptake and ensure equitable access to preventive services. These findings contribute to the ongoing discussions on public health strategies and underscore the necessity for community and healthcare provider engagement to improve cancer screening rates in this population.

## 1. Introduction

Cancer is a leading cause of morbidity and mortality worldwide, significantly impacting the aging population [1]. The World Health Organization (WHO) reports that the global burden of cancer is expected to rise, with an estimated 27 million new cases and 17 million cancer deaths annually by 2030, with a considerable proportion occurring among older adults [2]. This demographic shift underscores the necessity for effective cancer screening and early detection strategies, which are crucial for improving outcomes and reducing cancer-related mortality [3,4].

Cancer screening is a pivotal public health strategy, crucial for early detection and management, which can significantly enhance survival outcomes. In Saudi Arabia, however, the practice of regular screening is not as widespread as needed, affecting all segments of the population [5]. This lack of utilization stems from a complex interplay of factors, not just among older adults who are the focus of this study, but universally across all age groups. Cultural beliefs and social norms play a substantial role in this dynamic, influencing perceptions of and behaviors toward health care. In many instances, cultural stigma, misconceptions about the nature of screenings, and a general reluctance to seek preventive care unless symptoms are pervasive create significant barriers to effective cancer management [6]. By examining these issues within the context of older adults—who are at a higher risk yet significantly under-screened—this study sheds light on broader systemic and cultural challenges that could inform more targeted, inclusive public health interventions to improve screening uptake across the entire population [7].

Screening aims to identify cancer in asymptomatic individuals at an early stage when treatment can be more effective. Early detection has been shown to significantly improve survival rates for many cancers, such as breast, colorectal, and cervical cancers [8]. However, despite the benefits, screening uptake among older adults remains suboptimal. Several factors contribute to this gap, including age-related physiological changes, comorbidities, and the complexities of navigating the healthcare system [9,10].

The barriers to cancer screening in older adults can be broadly categorized into structural, psychological, and social factors [11]. Structural barriers include logistical challenges such as transportation issues, lack of accessibility to healthcare facilities, and inadequate insurance coverage [12]. Many older adults face mobility limitations that make it difficult to attend appointments or access necessary services [13]. Additionally, healthcare systems often lack the resources to effectively engage and support older patients, leading to missed opportunities for screening [14,15].

Psychological barriers also play a critical role in the decision-making process regarding cancer screening [16]. Fear of diagnosis, anxiety about potential procedures, and misconceptions about the risks and benefits of screening can deter older adults from seeking necessary care [17]. Many individuals may perceive cancer screening as a painful or invasive process, leading to avoidance behaviors that ultimately hinder early detection efforts [18].

Social factors, including the influence of family and social networks, also significantly affect screening behaviors. Older adults often rely on family members or caregivers for health-related decisions. If these individuals have misconceptions about screening or prioritize other health concerns, the older adult may not receive the encouragement needed to pursue screening [19]. Furthermore, cultural attitudes toward health and illness can vary widely among different ethnic groups, influencing beliefs about cancer screening and the importance placed on early detection [20].

The public health and primary care sectors play crucial roles in addressing barriers to cancer screening. Healthcare professionals in these fields are positioned to support older adults by navigating the complexities of the healthcare system and enhancing screening uptake [21]. Effective public health strategies, including patient education and community outreach, are essential to overcome the obstacles faced by this demographic. Understanding these barriers is crucial for tailoring interventions that can significantly improve cancer screening rates among older adults [22].

Recent studies highlight the need for tailored interventions to improve screening rates among older populations. For example, community-based initiatives that address mobility issues, provide transportation services, or offer education on the importance of screening have shown promise in increasing uptake [23]. Furthermore, engaging family members and caregivers in the screening process may enhance participation rates, as they can offer support and assistance in navigating healthcare services [24]. These barriers include complex administrative procedures, limited availability of localized services, and inadequate information dissemination, which together hinder the effective navigation of the healthcare landscape for many individuals [25]. This study provides insights into the reality that these systemic issues, compounded by cultural and social factors, deter not only older adults, who are the focus of this study, but various demographics, highlighting the need for systemic reforms. By addressing these navigational complexities, along with enhancing education and community engagement, health authorities can significantly improve the accessibility and effectiveness of cancer screening services across all sectors of society.

In addition to practical interventions, understanding the lived experiences of older adults regarding cancer screening is vital [26]. Qualitative research exploring the perceptions, beliefs, and attitudes of older adults toward screening can provide valuable insights into the barriers they face [27]. Such studies reveal that older adults often view screening through a lens of personal experience, influenced by previous health encounters, the health of peers, and societal narratives surrounding cancer [28]. By incorporating these perspectives, healthcare providers can develop more effective, patient-centered approaches to encourage screening.

This study aims to explore the barriers to cancer screening and early detection among older adults using a mixed-methods approach. By integrating both quantitative and qualitative data, the research seeks to provide a comprehensive understanding of the factors influencing screening behaviors. Identifying these barriers is crucial for informing oncology nursing practice and developing targeted interventions to enhance the accessibility and effectiveness of cancer screening programs.

## 2. Materials and Methods

### 2.1. Study Design

This study employed a mixed-methods design, integrating both quantitative and qualitative approaches to explore the barriers to cancer screening and early detection among older adults in Riyadh City, Saudi Arabia. The quantitative component aimed to assess the prevalence and types of barriers encountered by older adults regarding cancer screening, while the qualitative component sought to gain in-depth insights into their personal experiences, perceptions, and attitudes related to these barriers. This dual approach allows for a comprehensive understanding of the complex factors influencing screening behaviors among older adults.

### 2.2. Study Setting

The research was conducted at King Saud University-affiliated hospitals and primary healthcare centers in Riyadh City. These facilities were chosen due to their accessibility to a large population of older adults who regularly visit for routine health check-ups and management of chronic diseases. The selected healthcare centers are equipped with various screening services, making them ideal locations for assessing cancer screening practices. The diverse demographic characteristics of the Riyadh population, encompassing different socioeconomic backgrounds, enhance the study’s representativeness.

### 2.3. Study Population

The target population for this study included older adults aged 60 years and above, who regularly attended the healthcare centers associated with King Saud University. Participants were required to be fluent in Arabic, cognitively capable of providing informed consent, and willing to participate in both the quantitative and qualitative components of the study. This age group was selected due to their higher susceptibility to various cancers [29], necessitating targeted screening efforts. Furthermore, the study aimed to capture a broad spectrum of experiences and barriers that this demographic faces concerning cancer screening.

### 2.4. Sampling and Sample Size

A non-probability convenience sampling technique was employed to recruit participants for both the quantitative and qualitative phases of the study. The study aimed to enroll 100 participants for the quantitative component and 20 participants for the qualitative component.

1. **Quantitative Sample**: A sample size of 100 participants was determined to ensure sufficient statistical power for analysis and the ability to detect meaningful relationships between the identified barriers and screening uptake. Inclusion criteria for this phase encompassed individuals aged 60 and above who could complete self-reported questionnaires. Participants were recruited during their routine visits to the healthcare centers, ensuring a representative sample of older adults accessing health services. The selection of 100 participants for this study was determined by the need for statistical robustness and logistical feasibility. This sample size provides sufficient power to detect significant trends and relationships within the data, while also being manageable for in-depth qualitative and quantitative analysis. It ensures a diverse representation of Riyadh’s older adult population, balancing comprehensiveness with the practical constraints of time and resources. Additionally, this number aligns with similar studies in the field, supporting the generalizability of the findings within the targeted demographic.
2. **Qualitative Sample**: For the qualitative phase, semi-structured interviews were conducted with 20 participants, purposively selected from the quantitative sample based on their willingness to share detailed insights regarding their experiences with cancer screening. Data saturation was achieved after conducting semi-structured interviews with 20 participants. This was determined when additional interviews no longer yielded new themes or insights, indicating that the collected data were sufficient to reflect the range of experiences and barriers within the target population. This approach ensures that the themes developed are robust and representative of the study participants, thereby enhancing the reliability and validity of our findings. The process involved continuous comparison of data across all interviews, with iterative analysis conducted alongside data collection to confirm when saturation was achieved. Participants who reported a higher awareness of screening services or those who expressed varied experiences in their questionnaires were particularly targeted to provide rich, diverse data.

### 2.5. Data Collection Tools

#### 2.5.1. Quantitative Component

Quantitative data were collected using a validated self-report questionnaire designed to measure barriers to cancer screening among older adults.

1. **Barriers to Cancer Screening Scale (BCSS)**
  ○ The Barriers to **Cancer Screening Scale (BCSS)** was utilized to assess various obstacles that older adults encounter in accessing cancer screening services. The BCSS is a comprehensive tool that focuses on multiple dimensions of barriers, including:
    ▪ **Logistical Barriers**: These include challenges related to transportation, availability of screening facilities, clinic operating hours, and the cost of screenings. Items may include statements such as “I find it hard to get to the clinic because of transportation issues” or “I am unable to schedule an appointment at a convenient time”.
    ▪ **Psychological Barriers**: This category addresses emotional responses associated with screening, such as fear of results, anxiety about procedures, and a lack of perceived need for screening. Items may include “I feel anxious about what the screening results might show” or “I believe I do not need to get screened because I feel healthy”.
    ▪ **Social Barriers**: This dimension encompasses influences from family and community, including lack of support from family members or cultural beliefs that may discourage screening. Items may include “My family does not support my decision to get screened” or “I do not talk to my friends about cancer screening”.
  ○ The BCSS consists of **30 items** rated on a **5-point Likert scale** (1 = Strongly Disagree to 5 = Strongly Agree). Higher total scores reflect a greater perceived barrier to cancer screening.
  ○ Pilot testing in 5 participants reveals a Cronbach’s alpha value of **0.88**, indicating excellent internal consistency. Validation studies involved diverse populations, ensuring their applicability to the target demographic of older adults.

#### 2.5.2. Qualitative Component

For the qualitative phase, **semi-structured interviews** were conducted to explore participants’ experiences with cancer screening and the barriers they faced. This qualitative approach allowed for an in-depth understanding of personal experiences while providing the flexibility for participants to express their thoughts freely.

- **Interview Guide Development**: An interview guide was crafted based on a thorough review of the relevant literature on cancer screening barriers and older adults’ health behaviors. The guide incorporated both open-ended questions and prompts to facilitate discussions around key topics, including personal experiences, perceived barriers, and support systems influencing screening behaviors.
- **Sample Questions**: The interview guide included the following open-ended questions to encourage participants to share their insights:
  ○ “What challenges have you faced in accessing cancer screening services?”
  ○ “How do you feel about the importance of regular cancer screenings?”
  ○ “Can you share your experiences with healthcare providers regarding cancer screening?”
  ○ “What would encourage you to participate in screening programs?”
- **Interview Process**: Interviews were conducted in a comfortable and private environment, either within healthcare centers or at participants’ homes, based on their preferences. Each interview was designed to last between 30 and 60 min to allow participants ample time to discuss their experiences thoroughly. Audio recordings were taken with participants’ consent, and detailed field notes were taken to supplement the audio data.

### 2.6. Data Collection Procedure

#### 2.6.1. Quantitative Data Collection

The quantitative data collection process was conducted over a period of four weeks at King Saud University-affiliated hospitals and primary healthcare centers in Riyadh City. The following steps were undertaken to ensure an efficient and effective data collection process:

1. **Preparation**: Prior to the commencement of data collection, the research team held orientation sessions with healthcare staff at the selected facilities. These sessions aimed to explain the study’s objectives, methodology, and the importance of cancer screening among older adults. Healthcare staff were encouraged to identify potential participants who met the eligibility criteria, which included individuals aged 60 years and above who were fluent in Arabic and cognitively capable of providing informed consent.
2. **Recruitment of Participants**: Eligible older adults were approached in various settings, including waiting areas and consultation rooms during their routine healthcare visits. The research team provided each potential participant with a detailed overview of the study, outlining its purpose, the expected duration of participation, and the voluntary nature of their involvement. Participants were encouraged to ask questions to clarify any concerns about the study.
3. **Informed Consent**: After ensuring that potential participants understood the study’s objectives and procedures, informed consent was obtained from those willing to participate. This process involved providing participants with written consent forms, which included information on their rights, confidentiality, and the option to withdraw from the study at any time without penalty. Participants were given time to read the consent form and ask any questions before signing.
4. **Administration of Questionnaires**: Upon obtaining consent, participants were asked to complete the **Barriers to Cancer Screening Scale (BCSS).** The questionnaire was provided in Arabic to facilitate understanding. To ensure inclusivity, the research team offered assistance to individuals with low literacy levels or physical impairments, such as reading questions aloud and recording their responses. Special consideration was given to participants with varying literacy levels to ensure that all individuals could comprehend and accurately respond to the survey questions. Prior to the commencement of the study, a preliminary assessment of literacy was conducted through a brief, informal conversation with each participant, which helped gauge their reading comfort and ability.
5. **Data Collection Environment**: The research team ensured that data collection occurred in a private setting to maintain confidentiality and allow participants to complete the questionnaires without distractions. Participants were informed that their responses would be anonymous and that no identifying information would be recorded on the questionnaires.
6. **Time Commitment**: The average time required for participants to complete the questionnaires was approximately 20–30 min. After completion, the research team collected the questionnaires, checked them for completeness, and securely stored the data for further analysis. Participants were thanked for their involvement and reminded of their right to withdraw their data at any point during the study.

#### 2.6.2. Qualitative Data Collection

The qualitative data collection followed approximately one week after the quantitative phase. The procedure for the qualitative component included:

1. **Participant Selection**: From the pool of participants who completed the quantitative questionnaires, 20 individuals were purposively selected for semi-structured interviews, based on their willingness to share detailed insights regarding their experiences with cancer screening. Selection criteria also considered variability in responses to ensure a diverse representation of experiences and barriers.
2. **Scheduling Interviews**: Selected participants were contacted via telephone to schedule interviews at a time and place convenient for them. The research team emphasized the importance of confidentiality and the voluntary nature of participation in the qualitative interviews. For the participants who agreed to participate, a second appointment was scheduled after the completion of the quantitative data.
3. **Interview Environment**: Interviews were conducted in private settings, either at healthcare centers or at participants’ homes, depending on their preference. This flexibility aimed to create a comfortable environment for participants to discuss sensitive topics related to cancer screening.
4. **Conducting Interviews**: Before beginning each interview, participants were reminded of the study’s purpose and reassured about the confidentiality of their responses. Verbal and written consent for audio-recording the interviews was obtained from each participant. The semi-structured format allowed for flexibility, enabling interviewers to explore topics in depth while adhering to the prepared interview guide.
5. **Duration and Content of Interviews**: Each interview lasted between 30 and 60 min, providing participants with ample time to articulate their experiences and perspectives. The interview guide facilitated discussions on barriers to screening, personal experiences, and suggestions for improving screening uptake. Interviewers encouraged participants to elaborate on their responses and share anecdotes to enrich the data collected.
6. **Data Recording and Transcription**: All interviews were audio-recorded with participants’ consent. After each interview, the audio recordings were transcribed verbatim in Arabic. The research team also maintained detailed field notes to capture non-verbal cues and contextual information. The transcripts were later translated into English for analysis.
7. **Quality Assurance**: To ensure data quality, the research team cross-referenced interview transcripts with field notes to confirm the accuracy and completeness of the information captured during the interviews.

To ensure the rigor of our qualitative data analysis, we adopted methods aligned with the principles outlined by Lincoln and Guba, focusing on enhancing the credibility, transferability, dependability, and confirmability of our findings [30]. Key strategies included:

1. **Triangulation**: We employed methodological triangulation by integrating both quantitative and qualitative data, allowing for cross-validation where findings from one method could be corroborated by another.
2. **Member Checking**: To enhance credibility and validate the data, we conducted member checking by sending back the interview transcripts to participants. This allowed them to review the accuracy and completeness of the information, as well as to provide additional comments or corrections. This process not only confirmed the validity of the data but also enriched the findings through participants’ clarifications or additional insights.
3. **Thick Description**: Detailed descriptions of the research context and the participants’ experiences were provided, facilitating transferability. This allows other researchers to evaluate the extent to which the findings may be applicable in other contexts.
4. **Audit Trail**: An audit trail was maintained, documenting all research decisions, procedures, and reflections throughout the study. This included detailed notes on the data collection and analysis processes, which enhances dependability and allows for an external audit, if necessary.
5. **Reflexivity**: The research team engaged in reflexivity throughout the study, documenting our own biases, assumptions, and influences on the research process. This was essential to ensure that the findings were shaped by the participants’ experiences and narratives rather than the researchers’ preconceptions.

### 2.7. Data Analysis

The analysis of data from this study employed both quantitative and qualitative methods to comprehensively assess barriers to cancer screening among older adults. For the quantitative component, data were analyzed using SPSS software (Version 26). The initial step involved preparing the data by reviewing the completed Barriers to Cancer Screening Scale (BCSS) for completeness and accuracy, followed by coding the variables for efficient analysis. Descriptive statistics, including means, standard deviations, frequencies, and percentages, were calculated to summarize the demographic characteristics of the participants and the primary study variables. Bivariate analyses, including chi-square tests and *t*-tests, were utilized to explore the relationships between demographic factors and perceived barriers to screening. Pearson’s correlation coefficient was computed to assess the association between total scores on the BCSS and screening uptake, with a significance level set at *p* < 0.05. Additionally, multiple regression analysis was conducted to identify potential demographic predictors of barriers to screening and screening uptake, and adjusted according to variables such as age, gender, education level, and income. Missing data were addressed using the multiple imputation technique to retain as much information as possible in the analysis. For the qualitative component, thematic analysis was employed to analyze the semi-structured interviews. The research team engaged in a systematic process that included familiarization with the data, generating initial codes, and identifying themes that captured significant patterns in participants’ experiences. Each theme was carefully defined and named, with illustrative quotes integrated to contextualize the findings. The results from both quantitative and qualitative analyses were triangulated to provide a nuanced understanding of the barriers to cancer screening, enriching the insights gained and informing strategies for enhancing screening uptake among older adults in Riyadh City.

### 2.8. Ethical Considerations

Ethical considerations for this study were paramount to ensure the protection of participants and the integrity of the research process. Approval was obtained from the Institutional Review Board (IRB) at King Saud University (24-961) on 1 August 2024, ensuring that the study adhered to ethical guidelines governing research involving human subjects. Informed consent was obtained from all participants prior to data collection, ensuring that they were fully aware of the study’s purpose, procedures, and their right to withdraw at any time without penalty. Participants were assured of the confidentiality and anonymity of their responses, with all data being securely stored and only accessible to the research team. Additionally, the study emphasized the voluntary nature of participation, and any potential risks were minimized by providing a supportive environment for participants to express their thoughts and experiences regarding cancer screening. This ethical framework aimed to uphold the dignity and rights of all participants while contributing valuable insights to improve healthcare practices for older adults.

## 3. Results

### 3.1. Demographic Characteristics

A total of 100 participants were included in the quantitative analysis (Table 1). The sample consisted of older adults aged 60 and above who regularly attended primary healthcare centers in Riyadh City, Saudi Arabia. The mean age of participants was 66.3 years (SD = 4.2), with a range from 60 to 75 years. Among the participants, 53% were male (n = 53) and 47% were female (n = 47). Regarding education, 35% had no formal education (n = 35), 42% had completed secondary education (n = 42), and 23% had attained higher education (n = 23). In terms of employment status, 60% were retired (n = 60), while 40% were engaged in part-time or informal work (n = 40). Additional variables, including marital status and income level, were also assessed.

The results from the Barriers to Cancer Screening Scale (BCSS) (Table 2) indicated significant barriers encountered by the participants. The mean total score on the BCSS was 75.6 (SD = 12.3), suggesting that participants perceived considerable barriers to accessing cancer screening services. A breakdown of scores revealed that logistical barriers had a mean score of 26.4 (SD = 5.6), psychological barriers had a mean score of 24.1 (SD = 6.2), and social barriers had a mean score of 25.1 (SD = 5.9). Specific items on the BCSS highlight the multifaceted challenges faced by older adults in seeking cancer screening.

Pearson’s correlation coefficient (Table 3) was used to examine the relationship between perceived barriers to cancer screening and several demographic variables, including age, education levels, income levels, and employment status. The results indicated significant correlations: a negative correlation between the total BCSS score and education level (r = −0.37, *p* < 0.001), a positive correlation between the total BCSS score and age (r = 0.28, *p* < 0.01), and a negative correlation between the total BCSS score and income level (r = −0.25, *p* < 0.05). Furthermore, employment status showed a significant correlation with barriers; retired individuals reported higher perceived barriers compared to those engaged in part-time work (r = 0.30, *p* < 0.01).

A logistic regression analysis (Table 4) was performed to determine the predictors of perceived barriers to cancer screening. The model included age, gender, education level, income level, and employment status as predictors. The results indicated that education level significantly predicted the likelihood of experiencing barriers, with individuals who had higher education being less likely to report significant barriers (Odds Ratio = 0.65, *p* = 0.002). Age and gender were not significant predictors in this model (*p* = 0.1 and *p* = 0.42, respectively), while income level approached significance (*p* = 0.06), indicating a trend where higher income was associated with fewer perceived barriers. Employment status was also significant, suggesting that retired individuals faced more barriers compared to those working part-time. The Barriers to Cancer Screening Scale (BCSS) was employed to quantitatively assess the barriers faced by older adults in accessing cancer screening services. Each item on the BCSS is rated on a 5-point Likert scale, and the scores across 30 items were summed to yield a total score for each participant, which could range from 30 to 150.

### 3.2. Qualitative Findings

In the qualitative interviews, participants articulated their perceptions of the barriers they faced in accessing cancer screening services (Tabe 5). Three primary thematic categories emerged from the data: (i) accessibility challenges; (ii) psychological barriers; and (iii) social influences. These three themes are described and discussed in detail below.

**1. Accessibility Challenges**

Participants frequently expressed concerns about the physical and logistical barriers that hindered their access to cancer screening. This theme was characterized by frustrations over the difficulties they encountered in reaching healthcare facilities and the availability of screening services.

- **Transportation Issues**: Many participants described the challenges posed by inadequate public transportation and the costs associated with private transportation.

One participant shared: “Getting to the clinic is a real challenge for me. The buses don’t come frequently, and I can’t afford a taxi every time I need to go for a check-up. It makes me put off screening”. (Participant 3)

Another participant echoed this sentiment, saying: “If I could just hop on a bus that takes me directly to the screening center, it would be so much easier. But the route is complicated, and I often just don’t have the energy to deal with it”. (Participant 10)

- **Availability of Services**: Participants also highlighted the limited availability of cancer screening services at local healthcare facilities. Many expressed disappointment that screenings were not offered regularly or were only available at distant hospitals.

“I always have to wait for special campaigns to get screened. It’s frustrating because I know I need to do it more often, but it’s not accessible unless they have those events”, explained Participant 7.

Another participant added: “The clinics near my home don’t have the right equipment for screenings, so I have to travel far, and that’s just not feasible for me most days”. (Participant 12)

These accessibility challenges left many participants feeling discouraged and anxious about their health, contributing to delays in seeking cancer screenings and complicating their overall health management.

**2. Psychological Barriers**

The emotional and psychological barriers participants faced were another critical theme. Many expressed feelings of fear, anxiety, and denial regarding cancer screening procedures, which deterred them from seeking necessary screenings.

- **Fear of Diagnosis**: Participants reported significant anxiety about the possibility of receiving a cancer diagnosis, which influenced their decision to avoid screenings altogether.

One participant reflected: “I know I should go get screened, but the thought of what they might find terrifies me. I’d rather not know than face the possibility of a cancer diagnosis. It feels safer to stay in the dark”. (Participant 5)

Another participant shared a similar sentiment, stating: “Every time I think about making an appointment, I get this sinking feeling. I convince myself that I’m fine, and that stops me from going”. (Participant 9)

- **Denial and Avoidance**: The theme of denial emerged prominently, with many participants admitting to rationalizing their avoidance of screenings.

“Sometimes, I just tell myself that as long as I feel okay, I don’t need to worry about it. I think many older people like me do the same”, explained Participant 4.

Another participant remarked: “When I hear people talking about the need for screenings, I just dismiss it. It’s easier not to think about it than to face what could be”. (Participant 8)

These psychological barriers were deeply intertwined with participants’ personal experiences and societal perceptions of cancer, often leading to avoidance behaviors that further compromised their health.

**3. Social Influences**

The role of social influences emerged as a significant theme in the discussions. Participants highlighted how family and community attitudes toward cancer screening impacted their willingness to pursue these services.

- **Lack of Family Support**: Many participants expressed that a lack of encouragement from family members contributed to their hesitance to seek screenings.

One participant explained: “My family doesn’t really talk about cancer or screenings. They think it’s something that happens to other people. When I mention getting screened, they brush it off, and that makes me feel like I shouldn’t worry about it either”. (Participant 8)

Another participant shared: “When I tried to discuss my concerns about screening, my family dismissed it. They don’t understand why it’s important to me. I felt like I was alone in this”. (Participant 11)

- **Cultural Beliefs and Stigma**: Participants noted that cultural attitudes toward health and illness often discouraged discussions about cancer screening.

“In my culture, we don’t like to discuss health problems openly. It makes it hard to feel like it’s okay to seek help or talk about screenings. People think it’s a sign of weakness”, said Participant 2.

Another participant remarked: “There’s this belief that if you go for screenings, it means you’re expecting something bad. That pressure makes it difficult to even consider going”. (Participant 6)

These social influences left participants feeling isolated in their health decisions, further exacerbating the barriers to accessing cancer screening.

**Table 5:** Thematic Analysis of Qualitative Findings.

Theme	Subtheme	Description	Participant Quotes
**1. Accessibility Challenges**	Transportation Issues	Participants reported difficulties in accessing clinics due to inadequate transportation options.	“Getting to the clinic is a real challenge for me. The buses don’t come frequently”. (Participant 3)
			“If I could just hop on a bus that takes me directly to the screening center, it would be easier”. (Participant 10)
	Availability of Services	Limited availability of screening services at local healthcare facilities was a significant concern.	“I always have to wait for special campaigns to get screened. It’s frustrating”. (Participant 7)
			“The clinics near my home don’t have the right equipment for screenings”. (Participant 12)
**2. Psychological Barriers**	Fear of Diagnosis	Anxiety about receiving a cancer diagnosis led to avoidance of screenings.	“I’d rather not know than face the possibility of a cancer diagnosis”. (Participant 5)
			“I convince myself that I’m fine, and that stops me from making appointments”. (Participant 9)
	Denial and Avoidance	Participants rationalized their avoidance of screenings through denial and justification.	“As long as I feel okay, I don’t need to worry about it”. (Participant 4)
			“It’s easier not to think about it than to face what could be”. (Participant 8)
**3. Social Influences**	Lack of Family Support	A lack of encouragement from family members impacted participants’ decisions to pursue screenings.	“My family doesn’t really talk about cancer or screenings”. (Participant 8)
			“When I tried to discuss my concerns, my family dismissed it”. (Participant 11)
	Cultural Beliefs and Stigma	Cultural attitudes discouraged discussions about health and screenings.	“In my culture, we don’t like to discuss health problems openly”. (Participant 2)
			“There’s this belief that if you go for screenings, it means you’re expecting something bad”. (Participant 6)

## 4. Discussion

The findings of this study highlight critical barriers to cancer screening among older adults in Saudi Arabia, a country with a rapidly aging population and increasing cancer prevalence. National health strategies have increasingly focused on enhancing preventive services, yet our results indicate substantial gaps that need addressing.

Accessibility challenges emerged as a prominent theme in the qualitative findings. Participants frequently reported logistical difficulties related to transportation and the availability of screening services. These findings are consistent with the existing literature, which highlights transportation as a critical barrier to healthcare access, particularly for older adults [12,31]. Limited public transportation options can significantly hinder older adults’ ability to attend medical appointments, including cancer screenings [32]. Research suggests that older individuals often rely on family members or caregivers for transportation, and when these support systems are lacking, it can lead to missed appointments and delayed screenings [33]. Additionally, the availability of screening services is crucial; many participants expressed frustration over the limited options for screening at local healthcare facilities. This echoes findings from other studies indicating that insufficient access to screening facilities directly correlates with lower screening rates among older populations [34,35]. Similarly to our findings, recent studies have identified transportation and geographic accessibility as significant barriers to healthcare in Saudi Arabia [36]. The limited availability of localized screening services in rural or underserved areas exacerbates these challenges, aligning with national reports that highlight the need for more accessible healthcare infrastructure [37].

Psychological barriers were another significant theme identified in this study. Participants expressed feelings of fear and anxiety regarding cancer diagnoses, which deterred them from seeking necessary screenings. This aligns with existing research, demonstrating that fear of diagnosis is a common psychological barrier faced by individuals when considering cancer screening [38]. The apprehension surrounding potential outcomes can lead to avoidance behaviors, as individuals prefer to remain unaware of their health status rather than confront the possibility of a serious illness [39]. Furthermore, denial emerged as a critical aspect of participants’ experiences. Many rationalized their avoidance of screenings by convincing themselves that they were healthy or that there was no need for screening. This tendency to minimize health concerns is prevalent in older adults, as they may wish to avoid the discomfort associated with confronting potential health issues [40,41]. The psychological barriers noted in our study, particularly the fear of a cancer diagnosis, are echoed in broader societal attitudes within Saudi Arabia. Cultural stigmas surrounding cancer and misconceptions about its prognosis remain prevalent, impacting screening uptake [34]. Public health campaigns focusing on education and awareness could mitigate these fears [42].

Social influences also played a significant role in shaping participants’ attitudes toward cancer screening. The lack of support from family members was a recurrent theme, with many participants feeling discouraged by their families’ dismissive attitudes toward the importance of screenings. This finding resonates with research indicating that family dynamics and support systems significantly influence health-seeking behaviors in older adults [43,44]. When family members downplay the importance of screening, it can lead to reduced motivation among older adults to pursue these vital health services. Moreover, cultural beliefs and stigma surrounding cancer further complicate the screening landscape. Participants reported that cultural norms often discourage open discussions about health issues, which can perpetuate myths and fears surrounding cancer [45]. Such cultural barriers can create an environment where individuals feel isolated in their health decisions, leading to further avoidance of screening opportunities [46]. Family support plays a pivotal role in health decisions among older Saudi Arabian adults, as confirmed by our findings. The family unit significantly influences decisions on preventive health measures, often deterring or delaying screening due to prevailing health beliefs and priorities [47]. Enhancing family-oriented health education could therefore be a critical step in improving screening rates.

The interplay between these barriers suggests a need for tailored interventions that address the unique challenges faced by older adults. For instance, community-based programs that provide transportation assistance could help mitigate some of the logistical challenges highlighted by participants [48]. Additionally, incorporating educational initiatives that focus on reducing fear and misinformation about cancer screening could empower older adults to engage more proactively with their health [49]. These interventions should also involve family members and caregivers, to foster a supportive environment that encourages screening behaviors.

Furthermore, addressing the psychological barriers faced by older adults is essential. Health professionals can play a pivotal role in alleviating the fears associated with cancer screenings by providing clear, empathetic communication about the screening process and the importance of early detection [50]. By creating a supportive and informative environment, healthcare providers can help mitigate the anxiety that often accompanies the screening experience. Moreover, implementing psychosocial support programs that address the emotional and psychological needs of older adults could prove beneficial in encouraging screening uptake [51].

This study’s findings also underscore the importance of cultural competence in healthcare delivery. Providers should be trained to understand and respect the cultural beliefs of older adults, while also providing education that challenges harmful stereotypes and the stigma associated with cancer [52]. Creating culturally sensitive interventions that resonate with the values and beliefs of diverse populations may enhance the effectiveness of cancer screening programs [53].

In the context of Saudi Arabia, cultural factors significantly influence health behaviors, including cancer screening practices. Cultural norms and beliefs concerning health and illness deeply affect decisions to participate in screening programs. For instance, the stigma associated with a cancer diagnosis, gender-specific barriers, and the dominance of family decision making can deter individuals from engaging in preventive care measures. This study thoroughly investigates these cultural dimensions to better understand how they contribute to the underutilization of cancer screening services among older adults. By identifying and addressing these cultural barriers, health initiatives can be tailored to be more culturally sensitive and effective, potentially increasing screening uptake.

Moreover, involving community organizations in promoting cancer awareness and screening can create a more inclusive approach to healthcare. Collaborating with local leaders and organizations can help tailor messages and interventions to align with community values, ultimately increasing the likelihood of participation in screening programs [54]. A deliberate effort was made to integrate quantitative and qualitative data to provide a comprehensive understanding of the barriers to cancer screening faced by older adults in Saudi Arabia. The quantitative results identified significant statistical barriers, such as logistical issues and psychological fears, which were then explored in depth through qualitative interviews. This mixed-methods approach allowed for a nuanced interpretation of the data, where qualitative insights explained the ‘why’ and ‘how’ behind the patterns observed in the quantitative analysis. For instance, while the quantitative data revealed a high incidence of psychological barriers, the qualitative interviews provided detailed personal accounts that illustrated the underlying fears and misconceptions about cancer screening.

Furthermore, this study contributes new insights into the complex interplay of cultural, social, and systemic factors influencing cancer screening in Saudi Arabia. One novel finding is the significant role of familial influence and cultural stigma, which were more pervasive and influential than previously documented in the literature. Our study also uniquely highlights the specific logistical challenges faced by older adults in urban areas of Riyadh, which have been underexplored in existing research. By detailing these barriers, the study not only adds to the existing body of knowledge, but also proposes targeted interventions that are informed by both the quantitative prevalence and qualitative depth of these barriers.

These contributions underscore the value of a mixed-methods approach in public health research, particularly in culturally rich and diverse settings like Saudi Arabia, where understanding the subtleties of social and cultural dynamics is key to effective health intervention design.

### 4.1. Implications of the Study

The findings of this study have several important implications for healthcare policy, practice, and future research. First, understanding the multifaceted barriers faced by older adults in accessing cancer screening highlights the need for tailored interventions. Healthcare providers and policymakers should prioritize the development of community-based programs that address logistical challenges, such as transportation and accessibility to screening facilities. Implementing transportation assistance services and enhancing the availability of screening options in local healthcare centers could significantly improve access for older adults.

Additionally, the psychological barriers identified in this study necessitate the incorporation of supportive counseling and educational initiatives that target fear and anxiety related to cancer screenings. Training healthcare professionals to communicate effectively and empathetically about the screening process can help alleviate concerns and encourage older adults to engage in preventive care.

Furthermore, the role of family and community support emerged as a critical factor influencing screening behaviors. Therefore, community engagement strategies that involve family members and caregivers should be developed to foster a supportive environment that encourages health-seeking behaviors among older adults. By addressing these social influences, healthcare initiatives can enhance the willingness of older adults to pursue cancer screenings. To effectively increase cancer screening rates among older adults in Saudi Arabia, tailored public health interventions are required. These should include mobile screening units to address accessibility issues, targeted psychological support programs, and community engagement initiatives that involve family and societal leaders in health promotion.

Lastly, regarding the implications for nursing, this study delineates clear roles for different nursing specialties in promoting cancer screening based on our findings. Public health nurses are ideally positioned to lead community outreach and education efforts, as they often operate within community settings and are integral to preventive care initiatives. They can use the findings of this study to develop educational programs that specifically address the cultural and psychological barriers identified. General nurses in primary care settings can incorporate awareness of cancer screening into routine care visits for older adults, making discussions about screening a regular part of health assessments. Oncology nurses, who are typically more involved with patients who have already been diagnosed with cancer, can also advocate for and educate about the importance of early detection, especially for patients at high risk or with a family history of cancer. Each nursing group has a distinct, complementary role to play in enhancing the effectiveness of cancer screening programs, ensuring that interventions are appropriately targeted to overcome the identified barriers.

### 4.2. Limitations of the Study

Despite its contributions, this study has some limitations that should be acknowledged. Firstly, the study employed a convenience sampling method, which may limit the generalizability of the findings. Participants were recruited from specific healthcare facilities affiliated with King Saud University, which may not represent the broader population of older adults in Riyadh or other regions of Saudi Arabia. Future studies should aim for a more diverse sample that includes participants from various backgrounds and healthcare settings.

Secondly, the reliance on self-reported measures for assessing barriers to cancer screening may introduce bias, as participants might underreport or overreport their experiences. The subjective nature of self-reported data can lead to inaccuracies, particularly concerning psychological barriers. Incorporating objective measures or triangulating data with healthcare utilization records could strengthen the validity of the findings.

Additionally, the qualitative component involved a limited number of interviews (20 participants), which may not capture the full range of experiences and perceptions among older adults regarding cancer screening. Although data saturation was achieved, future research could benefit from a larger qualitative sample to gain deeper insights into the barriers faced by different subgroups within the older adult population.

Lastly, the cross-sectional design of the study limits the ability to draw causal inferences. Longitudinal studies are needed to examine how barriers to cancer screening may change over time and to evaluate the effectiveness of interventions designed to improve screening uptake.

## 5. Conclusions

In conclusion, this study provides valuable insights into the barriers faced by older adults in accessing cancer screening services. The findings highlight the interplay between the accessibility challenges, psychological barriers, and social influences that impact screening behaviors. Addressing these multifaceted barriers is essential for improving cancer screening rates and, ultimately, health outcomes among older adults.

The study emphasizes the need for tailored interventions that consider the unique challenges faced by this population, including logistical support, psychological counseling, and enhanced community engagement. By fostering an environment that promotes cancer screening and addresses the barriers identified in this study, healthcare systems can work toward ensuring that older adults have equitable access to life-saving preventive services. Future research should continue to explore these barriers and evaluate the effectiveness of targeted interventions to enhance cancer screening uptake in older adults across diverse settings.

## Figures and Tables

**Table 1:** Demographic Characteristics (n = 100).

Variable	Mean (SD) or %	n
Age (years)	66.3 (4.2)	100
Gender		
Male	53%	53
Female	47%	47
Education		
No formal education	35%	35
Secondary education	42%	42
Higher education	23%	23
Employment status		
Retired	60%	60
Part-time/informal work	40%	40
Marital Status		
Married	65%	65
Single	20%	20
Widowed	15%	15
Income Level		
Low (<5000 SAR)	40%	40
Medium (5000–10,000 SAR)	45%	45
High (>10,000 SAR)	15%	15

**Table 2:** Barriers to Cancer Screening (n = 100).

Barrier Type	Mean (SD)	% Agree (≥4)	Range
Logistical Barriers	26.4 (5.6)	75%	12–36
Psychological Barriers	24.1 (6.2)	68%	10–35
Social Barriers	25.1 (5.9)	50%	11–36
Total BCSS Score	75.6 (12.3)	-	50–102

**Table 3:** Correlation Analysis (n = 100).

Variable	r	*p*-Value
BCSS Total Score and Age	0.28	<0.01
BCSS Total Score and Education Level	−0.37	<0.001
BCSS Total Score and Income Level	−0.25	<0.05
BCSS Total Score and Employment Status	0.30	<0.01

**Table 4:** Logistic Regression Analysis for Barriers to Cancer Screening.

Predictor	B	SE	Odds Ratio (OR)	95% CI	*p*-Value
Education Level	−0.43	0.13	0.65	0.50–0.85	0.002
Age	0.05	0.04	1.05	0.97–1.13	0.1
Gender	0.10	0.25	1.10	0.65–1.89	0.42
Income Level	−0.24	0.13	0.78	0.61–1.01	0.06
Employment Status	0.37	0.16	1.45	1.05–2.01	0.03

## Data Availability

All data are available within the manuscript.
